# Adnexal Torsion of a Luteinized Ovarian Cyst in Early Pregnancy: A Case Report and Literature Review

**DOI:** 10.7759/cureus.114494

**Published:** 2026-08-13

**Authors:** Houda Lemouedden, Chadia Khalloufi, Houda Moustaide, Saad Benkirane, Ghizlane Erradi

**Affiliations:** 1 Department of Gynecology and Obstetrics, Mohammed VI University Hospital, Faculty of Medicine and Pharmacy of Tangier, Abdelmalek Essaadi University, Tangier, MAR; 2 Department of Anatomopathology, Mohammed VI University Hospital, Faculty of Medicine and Pharmacy of Tangier, Abdelmalek Essaadi University, Tangier, MAR

**Keywords:** adnexal torsion, doppler ultrasound, early pregnancy, fertility preservation, luteinized ovarian cyst

## Abstract

Ovarian torsion is a gynecological emergency that may occur during pregnancy, particularly during the first trimester, when its symptoms often mimic benign gestational complaints, making diagnosis challenging. Early recognition and timely management are essential to preserve ovarian function and ensure favorable maternal outcomes. We report the case of a 23-year-old primigravida at eight weeks and two days of amenorrhea who presented to the obstetric emergency department with acute pelvic pain without vaginal bleeding. Clinical examination revealed a closed cervical os with no evidence of bleeding. Pelvic ultrasound demonstrated a left ovarian cyst complicated by adnexal torsion. Due to persistent pain, an urgent mini-laparotomy was performed within five hours of symptom onset. Intraoperative findings confirmed adnexal torsion, and conservative surgical management consisting of detorsion and cystectomy was successfully achieved with preservation of the ovary. The postoperative course was uneventful, with ongoing favorable pregnancy evolution. This case highlights the increased risk of ovarian torsion during early pregnancy due to hormonal and anatomical changes, particularly the presence of corpus luteum cysts and increased adnexal mobility. Because clinical presentation is often nonspecific, ultrasound with Doppler remains the imaging modality of choice. Prompt surgical intervention should not be delayed, and ovarian preservation is frequently feasible and desirable in young patients to maintain future reproductive potential.

## Introduction

Adnexal torsion is a time-sensitive gynecologic emergency resulting from the rotation of the ovary and/or fallopian tube around its vascular pedicle, compromising blood supply and threatening ovarian viability. Although rare during pregnancy, with an estimated incidence of one to five cases per 10,000 gestations, it remains a significant diagnostic consideration, accounting for 2.7% to 7.4% of gynecological emergencies [1,2]. If left untreated, torsion may lead to ischemia, necrosis, loss of ovarian function, and pregnancy complications.

Pregnancy itself increases the risk of torsion, particularly during the first trimester. This is due to increased ligamentous laxity, enhanced adnexal mobility, and the frequent presence of corpus luteum or luteinized cysts [1]. Other predisposing factors include ovarian hyperstimulation syndrome (OHSS) and assisted reproductive technologies (ART), both of which are associated with multiple large follicular cysts [1]. The clinical presentation, typically consisting of progressive lower abdominal pain, nausea, vomiting, and signs of peritoneal irritation, is often indistinguishable from that of appendicitis, renal colic, ectopic pregnancy, or a ruptured ovarian cyst, which complicates the diagnostic process [2]. 

Among adnexal masses during pregnancy, corpus luteum cysts are the most common, accounting for 13% to 17% of cases [3]. These cysts usually regress spontaneously by the second trimester, when the placenta assumes the role of progesterone production. However, surgical removal of an active corpus luteum during early pregnancy may result in miscarriage if not followed by adequate hormonal support [4]. Other adnexal lesions encountered during pregnancy include hemorrhagic cysts, follicular cysts, benign teratomas, ovarian fibromas, and serous cystadenomas. Cyst size does not always predict the risk of torsion, as even small cysts can lead to this complication in early pregnancy [5]. 

We report a case of adnexal torsion associated with a small luteinized ovarian cyst during early spontaneous pregnancy. The educational value of this case lies in the occurrence of torsion despite the small size of the cyst, which measured approximately 3 cm, emphasizing that cyst size alone should not lower clinical suspicion when persistent severe pelvic pain and suggestive Doppler findings are present. Management required balancing urgent surgical exploration with the preservation of ovarian function and pregnancy viability. The case also documents successful conservative detorsion and cystectomy through mini-laparotomy when emergency laparoscopy was unavailable, together with luteal support following excision of a luteinized ovarian cyst. In this patient, timely surgical management restored ovarian viability while maintaining the ongoing pregnancy.

## Case presentation

A 23-year-old primigravida with no relevant medical or surgical history presented during a spontaneous pregnancy at eight weeks and two days of gestation. She had no known risk factors for ectopic pregnancy, including previous pelvic inflammatory disease, endometriosis, tubal surgery, or use of ART. She reported acute, severe left-sided pelvic pain that persisted despite oral paracetamol and antispasmodic treatment. She had no vaginal bleeding, nausea, vomiting, urinary symptoms, or gastrointestinal complaints.

The patient was conscious and afebrile, with stable cardiovascular and respiratory status on initial assessment. Her blood pressure was 120/60 millimeters of mercury, her respiratory rate was 18 per minute, and her body temperature was 37 degrees Celsius. She described her abdominal pain as intense, rating it seven out of ten on the pain scale. On abdominal examination, there was localized tenderness in the left iliac fossa, without guarding, rebound, or abdominal distension. No masses were palpable, and bowel sounds were normal. Gynecological examination, including speculum inspection and bimanual palpation, revealed a closed, violaceous cervix consistent with pregnancy, with no evidence of bleeding. The uterus size was consistent with early pregnancy, and there were no adnexal masses, cervical motion tenderness, or signs of peritoneal irritation. The absence of a palpable adnexal mass did not exclude torsion and may be explained by the deep pelvic location of the ovary and the limited sensitivity of bimanual examination for detecting adnexal abnormalities. 

She was admitted for close observation and pain management. Given the persistence of severe pain despite initial oral analgesia, intravenous morphine was initiated under supervision. This decision was based on the intensity of symptoms and the need to ensure maternal comfort while further investigations were underway.

Initial laboratory investigations are summarized in Table 1. The white blood cell count was mildly elevated at 11,110 cells/µL, with a hemoglobin level of 11.6 g/dL, a platelet count of 211,000 cells/µL, and a normal C-reactive protein level of 1 mg/L.

**Table 1:** Laboratory findings on admission

Parameter	Result	Reference Range
White Blood Cell Count (cells/µL)	11,110	4,000–10,000
Hemoglobin (g/dL)	11.6	12.0–16.0
Platelet Count (cells/µL)	211,000	150,000–400,000
C-reactive Protein (mg/L)	1	<5

**Figure 1:**
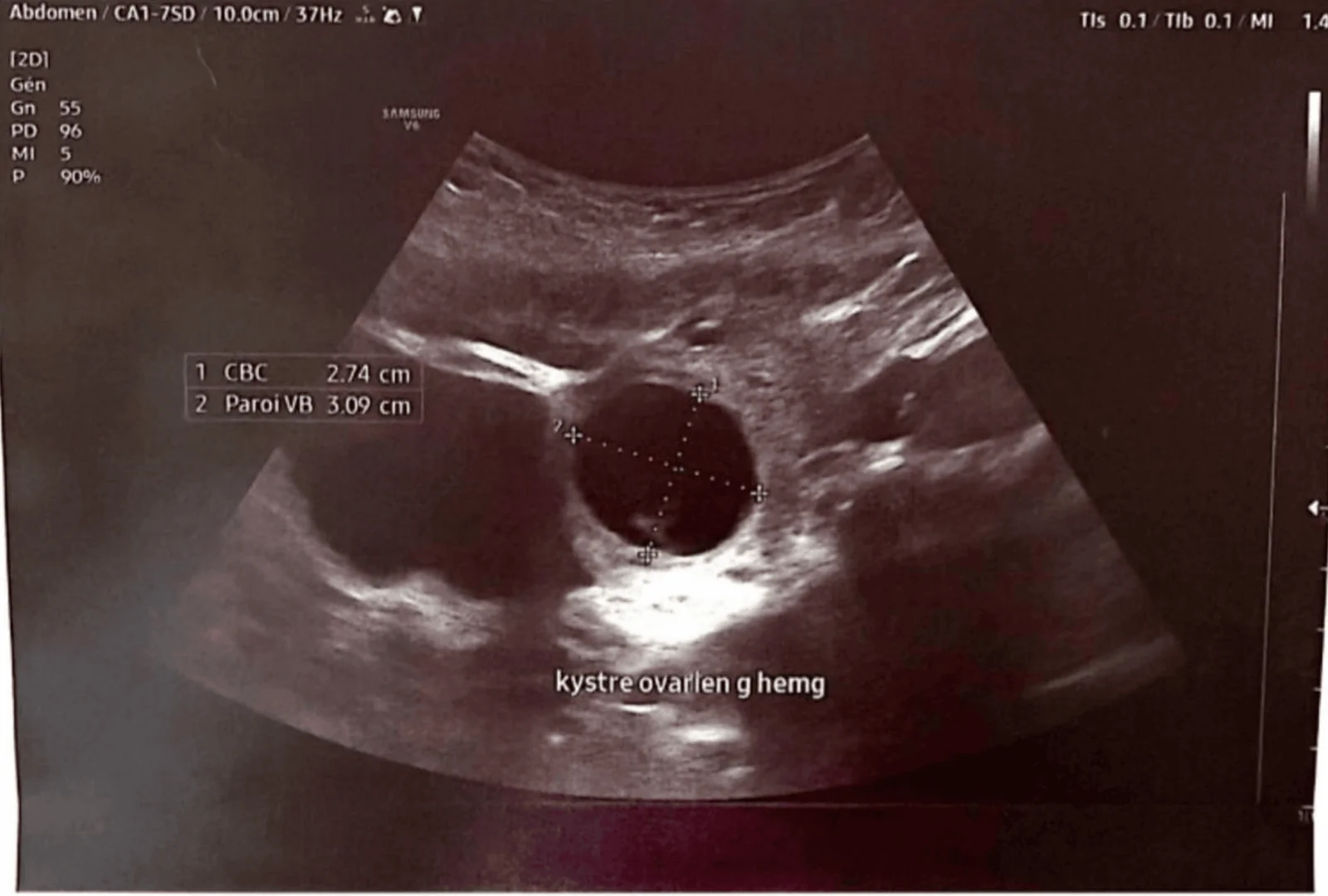
Suprapubic ultrasound showing a left ovarian cyst Suprapubic ultrasound image showing a left ovarian cyst measuring approximately 2.74 × 3.09 cm. The cyst appears unilocular and anechoic, with a thin wall and posterior acoustic enhancement, consistent with a functional ovarian cyst. Translation of text at the bottom of the figure: “Left hemorrhagic ovarian cyst." Other text visible on the figure: "CBC" refers to the largest cyst measurement; “Paroi VB" refers to the bladder wall.

Pelvic ultrasound revealed an intrauterine pregnancy with fundal implantation, showing visible fetal cardiac activity and corresponding to a gestational age of approximately eight weeks of amenorrhea, with a crown-rump length (CRL) of 17 millimeters. A small subchorionic hematoma was also noted adjacent to the gestational sac, located in the same fundal region. The right ovary looked normal, but the left one was clearly enlarged, about 70 by 48 millimeters, with a hyperechoic stroma measuring 14 mm. No vascular flow was detected on Doppler examination. The whirlpool sign was not documented during the initial ultrasound examination. Inside, there was one cyst measuring 27 by 30 mm. These findings strongly pointed towards left adnexal torsion. A small amount of free fluid was also found in the Douglas pouch. The left ovarian cyst identified on ultrasound is illustrated in Figure 1. 

The main differential diagnoses considered were a hemorrhagic or ruptured ovarian cyst, heterotopic pregnancy, renal colic, and appendicitis. The visualization of a viable intrauterine pregnancy without a separate adnexal gestational sac, the absence of vaginal bleeding, urinary symptoms, or gastrointestinal complaints, and the presence of an enlarged avascular left ovary favored adnexal torsion. 

As the pain persisted and progressively worsened despite strong opioid analgesia, surgical exploration was indicated and performed five hours after symptom onset. The patient was informed preoperatively about the potential need for oophorectomy depending on the intraoperative findings. Although laparoscopy is generally the preferred surgical approach for adnexal torsion during pregnancy, it was unavailable in our emergency setting. To avoid delaying surgical exploration in the presence of persistent severe pain and Doppler findings suggestive of torsion, mini-laparotomy was selected to allow prompt diagnosis and conservative treatment whenever feasible.

Operative technique

**Figure 2:**
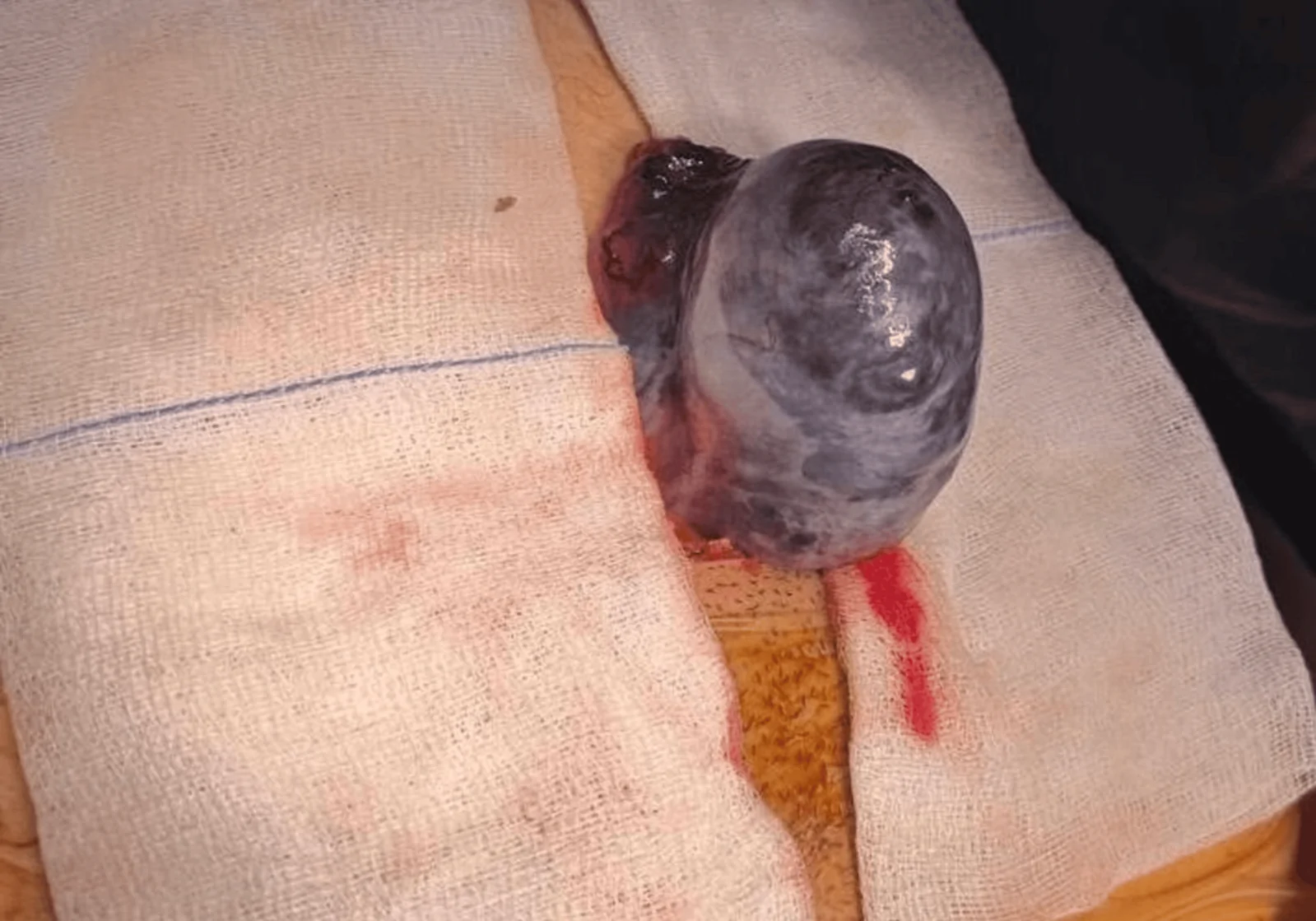
Intraoperative view of the left ovary immediately after detorsion Intraoperative image of the left ovary, temporarily exteriorized through the mini-laparotomy incision, immediately after detorsion. The ovary is markedly enlarged and presents a bluish-black coloration with a tense and smooth surface.

**Figure 3:**
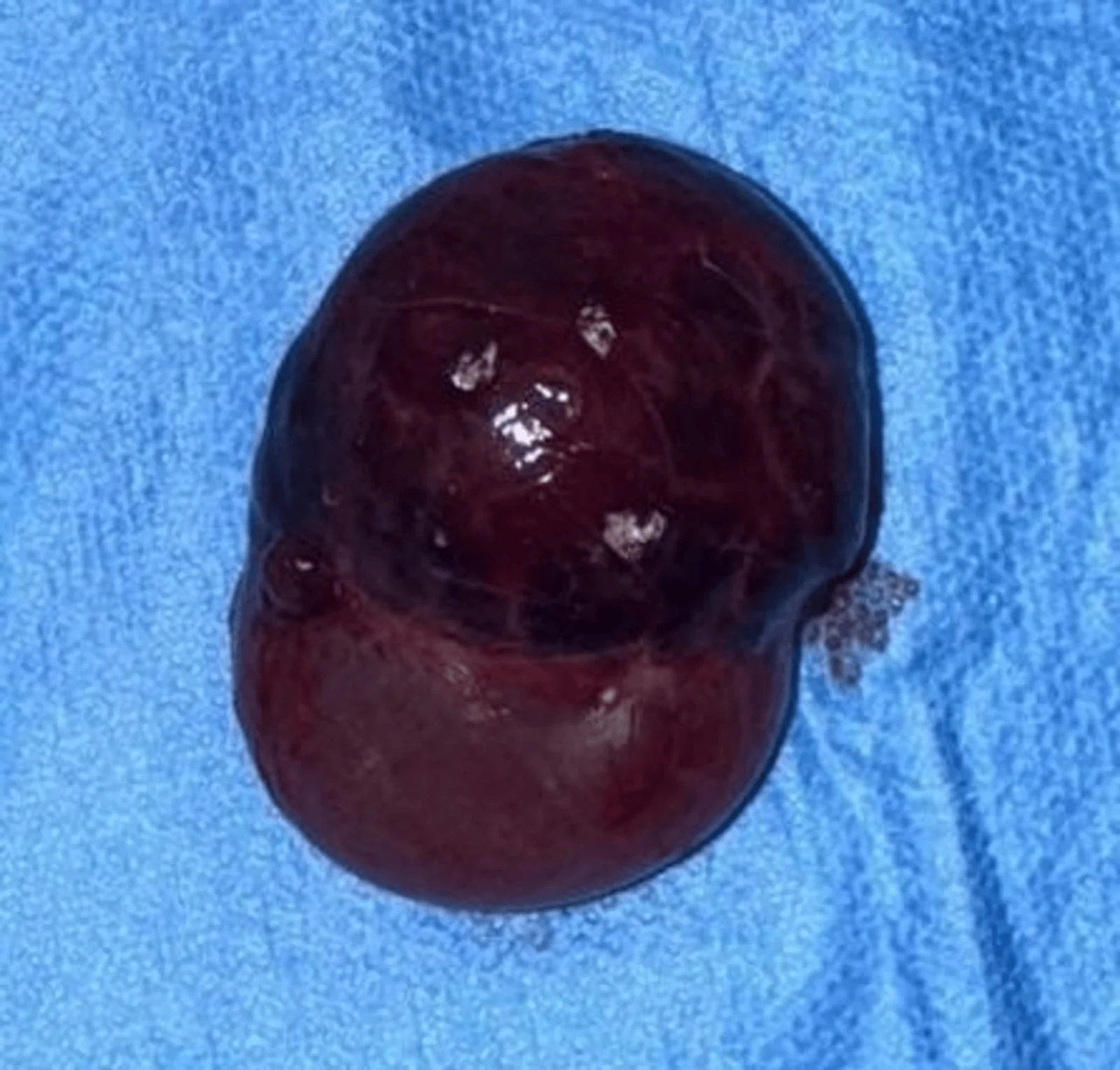
Excised ovarian cyst after cystectomy Image of the operative specimen following ovarian cystectomy, showing a smooth, congestive external surface and dark reddish coloration.

The procedure was performed under spinal anesthesia via a mini Pfannenstiel incision. No signs of peritoneal irritation were observed upon entry. Intraoperative findings revealed a twisted, bluish, cystic ovarian mass on the left side with two complete turns of the pedicle. The cyst was thin-walled, unilocular, and without septations and measured approximately 30 mm. The contralateral adnexa and the uterus appeared normal, with no evidence of carcinomatosis. Detorsion of the adnexa was successfully performed, followed by ovarian cystectomy. Reperfusion was achieved, and the ovary progressively regained a viable pink coloration. The peritoneal cavity was irrigated, and cytological samples were obtained. The excised specimen was sent for histopathological examination. Figure 2 shows the intraoperative appearance of the ovary immediately after detorsion, while Figure 3 depicts the excised ovarian cyst after cystectomy.

**Figure 4:**
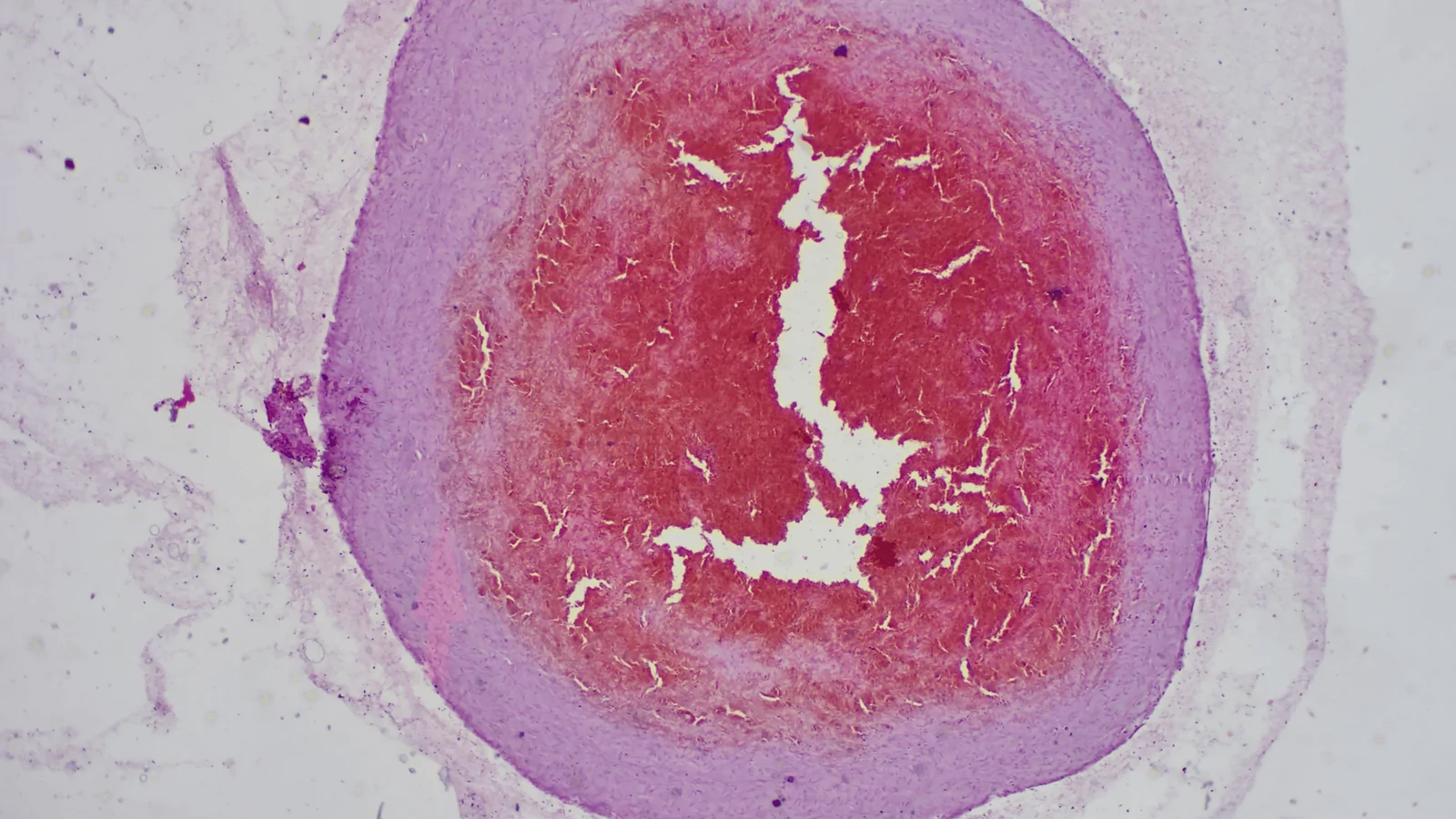
Histopathological appearance of the luteinized ovarian cyst Microscopic section showing a cystic cavity filled with fibrino-hemorrhagic material, consistent with hemorrhagic changes secondary to adnexal torsion (hematoxylin and eosin stain, original magnification ×10).

**Figure 5:**
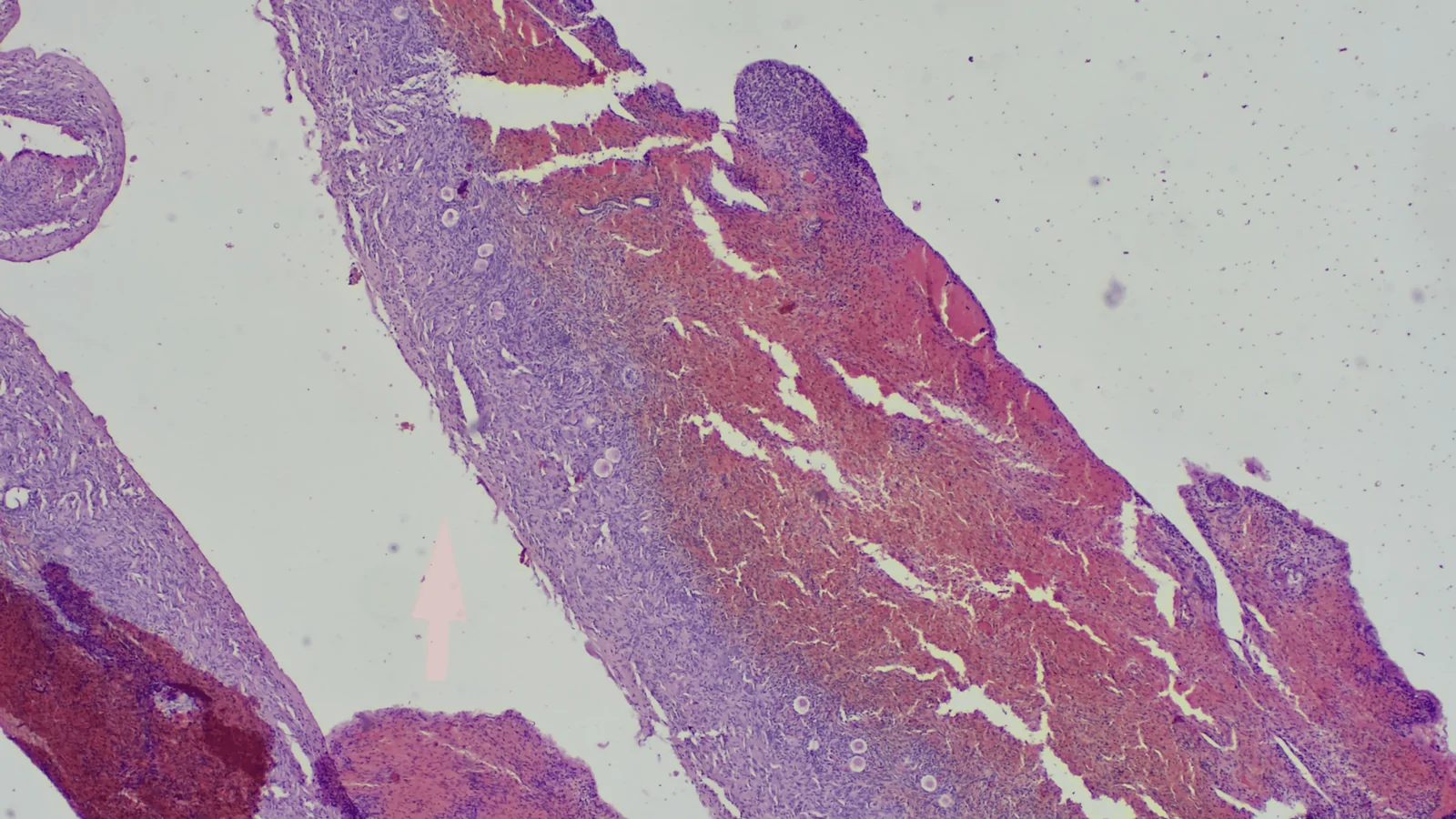
Histopathological changes in ovarian stroma after adnexal torsion Microscopic section showing ovarian stroma with extensive hemorrhagic suffusion and necrotic foci, consistent with hemorrhagic infarction secondary to adnexal torsion (hematoxylin and eosin stain, original magnification ×10).

Histopathological examination revealed a luteinized ovarian cyst with hemorrhagic and necrotic changes related to adnexal torsion. Microscopic examination showed a cystic cavity filled with fibrino-hemorrhagic material (Figure 4) and ovarian stroma with extensive hemorrhagic suffusion and necrotic foci (Figure 5). No evidence of malignancy was identified.

The postoperative recovery was uneventful. The patient received standard postoperative care, including analgesics, antibiotic prophylaxis, and thromboprophylaxis. She had been regularly taking folic acid since the beginning of her pregnancy. Because cystectomy involved a luteinized ovarian cyst at eight weeks of gestation, vaginal progesterone at a dose of 400 mg twice daily was initiated perioperatively as luteal support and continued until 16 weeks of gestation. Obstetrical ultrasound examinations performed before and after surgery confirmed ongoing fetal viability. No postoperative complications were noted. The patient remains under regular follow-up in our outpatient clinic with no abnormalities observed to date.

## Discussion

Adnexal torsion represents a critical surgical emergency during pregnancy. According to Hasson et al. over half (54.6%) of torsion cases in pregnant women are diagnosed during the first trimester, likely due to increased adnexal mobility while the uterus is still relatively small [6]. Functional ovarian cysts, particularly corpus luteum cysts, are frequently observed in early gestation and may contribute to the risk of torsion.

Torsion tends to occur more commonly on the right side, a pattern attributed to the longer utero-ovarian ligament on the right and the stabilizing effect of the sigmoid colon on the left [7]. In our case, however, torsion involved the left ovary.

Typical clinical signs of adnexal torsion include sudden, localized pelvic pain often accompanied by gastrointestinal or urinary symptoms. However, during pregnancy, these signs may be subtle, variable, or even absent, making diagnosis particularly challenging. In our case, the patient presented with acute left-sided pelvic pain unrelieved by simple analgesics in the absence of digestive or urinary complaints. While this presentation raised clinical suspicion, it remained nonspecific. In a retrospective study spanning 10 years, Ginath et al. analyzed 262 cases of ovarian torsion, including 64 pregnant and 198 non-pregnant women. Abdominal pain was nearly universal in both groups (97% vs. 96%). Nausea and vomiting occurred in 42% of pregnant patients and 48% of non-pregnant patients (p = 0.28), while fever was uncommon in both groups (5% vs. 11%, p = 0.33). Rates of abdominal tenderness (89% vs. 85%, p = 0.50) and right-sided involvement (66% vs. 61%, p = 0.75) were also comparable. Notably, the time from symptom onset to hospital admission was significantly shorter in pregnant women (mean 12.0 ± 18.5 hours vs. 31.2 ± 34.9 hours, p < 0.001), possibly reflecting heightened vigilance due to fetal concerns [8]. These overlapping and nonspecific clinical features highlight the diagnostic ambiguity of adnexal torsion in pregnancy, underlining the importance of timely imaging and surgical evaluation when clinical suspicion arises.

In early pregnancy, a series of hormonal and anatomical changes silently increases the risk of adnexal torsion. Elevated levels of human chorionic gonadotropin (hCG) stimulate the growth of corpus luteum cysts and increase ovarian volume, potentially altering the position of the ovary. Concurrently, the enlarging uterus disrupts the normal pelvic anatomy, increasing adnexal mobility and predisposing the ovary to twist around its vascular pedicle. Once torsion occurs, venous outflow is typically compromised first, resulting in congestion and stromal edema. If uncorrected, arterial inflow is eventually reduced, leading to ischemia and, ultimately, necrosis of the ovarian tissue [7].

The differential diagnosis of adnexal torsion includes ectopic or heterotopic pregnancy, appendicitis, renal colic, hemorrhagic or ruptured ovarian cyst, tubo-ovarian abscess, endometriosis, and degenerating fibroids. In pregnant patients presenting with acute severe pelvic pain, the initial assessment should establish maternal hemodynamic stability and exclude an immediate obstetric emergency. This should be followed by a focused history and physical examination, targeted laboratory investigations, and pelvic ultrasound with Doppler as the first-line imaging modality. When ultrasound findings are inconclusive and the patient is clinically stable, magnetic resonance imaging may provide additional diagnostic information. However, persistent severe pain associated with ovarian enlargement or abnormal Doppler flow should prompt urgent surgical evaluation because no individual clinical, laboratory, or imaging finding can definitively confirm or exclude torsion preoperatively.

Laboratory findings should be considered supportive rather than diagnostic. In a retrospective study by Melcer et al., a white blood cell count above 11,000 cells/mm³ was observed in 30.9% of pregnant women with adnexal torsion, compared with 56.5% of non-pregnant patients [9]. Mild leukocytosis may also occur physiologically during pregnancy and therefore lacks specificity. In our patient, the white blood cell count was mildly elevated at 11,110 cells/µL, while the C-reactive protein level was normal. These findings neither confirmed nor excluded torsion and were interpreted in conjunction with the persistent symptoms and Doppler findings.

Pelvic ultrasound with Doppler remains the first-line imaging modality for evaluating adnexal torsion during pregnancy, owing to its safety, availability, and non-ionizing nature, while grayscale ultrasound may reveal nonspecific features such as ovarian enlargement, peripheral follicular distribution, or the presence of an associated cyst. Doppler imaging is essential for assessing ovarian vascular flow. In a cross-sectional study conducted between 2011 and 2012, Niafar et al. evaluated 323 women under the age of 40 presenting with acute pelvic pain and clinical suspicion of adnexal torsion. All patients underwent transabdominal ultrasound followed by laparotomy within six hours. Among the 28 pregnant women (8.7%) included, adnexal torsion was significantly more frequent compared to non-pregnant women (35.7% vs. 11.1%; p < 0.001). Absence of ovarian blood flow was observed in 72.1% of surgically confirmed torsion cases. The diagnostic performance of Doppler ultrasound demonstrated a specificity of 99.6%, a positive predictive value (PPV) of 96.9%, a negative predictive value (NPV) of 95.9%, and an overall accuracy of 96% with a kappa coefficient of 0.84 compared to surgical findings [10]. In our patient, the absence of vascular flow on Doppler imaging supported the diagnosis and was subsequently confirmed during surgery.

Alternative imaging techniques such as magnetic resonance imaging (MRI) and computed tomography (CT) may be considered when ultrasound results are inconclusive or atypical. CT is seldom used during pregnancy due to ionizing radiation exposure but may assist in diagnosis in selected emergency situations. MRI, by contrast, provides excellent soft tissue contrast without radiation and has demonstrated high diagnostic performance for adnexal torsion. Characteristic MRI findings include stromal edema, peripheral follicular distribution, ovarian hemorrhage, twisted vascular pedicle (whirlpool sign), and tubal wall thickening greater than 10 mm. A 2021 systematic review and meta-analysis found that MRI had a pooled sensitivity of 0.81 (95% CI: 0.63-0.91) and specificity of 0.91 (95% CI: 0.80-0.96) in detecting adnexal torsion, outperforming CT in identifying complex adnexal pathology [11]. In our case, MRI was not performed, as ultrasound findings and clinical presentation were sufficient to indicate surgical exploration.

Current surgical management of adnexal torsion favors detorsion and ovarian preservation whenever technically feasible, particularly in young patients and during pregnancy. In a retrospective study by Feng et al. including 109 women with surgically confirmed adnexal torsion, 25 of whom were pregnant, conservative surgery was performed in 50% of pregnant women with neoplastic lesions and 42.1% of those with non-neoplastic lesions. Among non-pregnant women, the corresponding rates were 58.8% and 18.2%, respectively [12]. These findings reflect the priority given to ovarian preservation during pregnancy, particularly for benign-appearing lesions.

The initial bluish or black appearance of the ovary should not alone justify adnexectomy because gross coloration does not reliably predict irreversible loss of ovarian function. After detorsion, the adnexa should be reassessed for restoration of perfusion and structural integrity. Cystectomy may be performed when a benign-appearing cyst can be safely separated while preserving ovarian tissue. Adnexectomy should be reserved for exceptional situations, including a mass strongly suspicious for malignancy, structurally unsalvageable or disintegrating adnexal tissue, or hemorrhage that cannot be controlled conservatively. In our patient, the mass appeared benign, the ovarian tissue remained structurally intact, and the ovary progressively regained a viable pink coloration after detorsion. These findings supported cystectomy with ovarian preservation rather than adnexectomy.

Ovariopexy, or surgical fixation of the ovary, is not routinely recommended after a first episode of adnexal torsion because the risk of recurrence is generally low and evidence regarding its effectiveness remains limited. It may be considered in selected patients with recurrent torsion, a solitary remaining ovary, or a persistent anatomical predisposition such as an excessively elongated utero-ovarian ligament. The decision should be individualized because fixation requires additional surgical manipulation and may alter the normal tubo-ovarian relationship. In a 2022 case report by Jalal et al. involving a 14-week pregnant patient with adnexal torsion, detorsion was performed without ovariopexy, with an uneventful postoperative course [13]. In our patient, this was the first episode of torsion; the contralateral adnexa was normal, and no anatomical risk factor for recurrence was identified. Ovariopexy was therefore not performed.

Both laparoscopy and laparotomy are utilized in the surgical management of adnexal torsion during pregnancy, with the choice depending on gestational age, surgical expertise, resource availability, and patient stability. In a retrospective study by Wu et al., which included 105 women with surgically confirmed adnexal torsion (33 pregnant and 72 non-pregnant), laparotomy was the predominant approach among pregnant patients (84.8%), whereas laparoscopy was more commonly employed in non-pregnant women (54.2%) [14]. These findings reflect differences in practice patterns and resource availability but do not establish the superiority of laparotomy during pregnancy. In our emergency setting, laparoscopy was unavailable. Mini-laparotomy was therefore performed within five hours of symptom onset to avoid delaying surgical exploration and to permit prompt detorsion and ovarian-preserving cystectomy.

Histopathological evaluation is crucial to determine the nature of the torsed adnexa and to guide surgical management. In a retrospective study conducted over a 10-year period, Seo et al. analyzed 239 women with surgically confirmed adnexal torsion, including 33 pregnant and 206 non-pregnant patients. Corpus luteum cysts were more frequently found in pregnant women (42.4%) than in non-pregnant patients (26.7%). Conversely, dermoid cysts were more common in non-pregnant women (29.6% vs. 6.1%). Serous cysts were identified in 17.0% of non-pregnant patients and 6.1% of pregnant ones. Mucinous cysts were reported in 10.2% and 9.1%, respectively. Other lesions, such as inflammatory, endometriotic, and metastatic cysts, were infrequent in both groups. Notably, no malignant lesions were observed in any of the pregnant patients. A higher proportion of cases without histological biopsy was recorded among pregnant women (18.2% vs. 1.9%), likely reflecting a conservative intraoperative approach focused on maternal-fetal safety [15]. In our case, histopathological examination revealed a torsed luteinized ovarian cyst without evidence of malignancy. This observation is consistent with the findings of Seo et al., confirming that functional and non-neoplastic cysts represent the most frequent cause of adnexal torsion during early pregnancy. These data further support the conservative surgical management adopted in our patient.

Postoperative complications following surgery for adnexal torsion during pregnancy are rare but clinically significant. In a 10-year retrospective study by Dvash et al. involving 94 pregnant women who underwent laparoscopic management, the miscarriage rate was 6.5%, mostly occurring within three weeks postoperatively during the first trimester. The preterm birth rate was 19.4%, with a notably higher incidence among women with twin pregnancies or those who conceived through ART [16]. No significant maternal or fetal morbidity was reported, confirming the favorable obstetric outcomes associated with laparoscopic management. In our case, the postoperative course was uneventful, and follow-up consultations have confirmed a favorable evolution of the pregnancy.

The corpus luteum plays a critical role in maintaining early pregnancy through progesterone secretion before completion of the luteoplacental transition. Hatata et al. described a similar case in which surgical removal of the corpus luteum was followed by immediate high-dose progesterone supplementation, with a favorable pregnancy outcome [17]. However, evidence from an isolated case report cannot establish the effectiveness of this intervention.

In the PRISM (PRogesterone In Spontaneous Miscarriage) trial, vaginal micronized progesterone at a dose of 400 mg twice daily was administered until 16 weeks of gestation to women presenting with early pregnancy bleeding. The greatest benefit was observed among women with a history of previous miscarriages [18]. However, that population differs from patients undergoing surgical excision of a luteinized ovarian cyst, and the findings cannot be directly extrapolated to this situation. In our patient, cystectomy was performed at eight weeks of gestation and involved a luteinized ovarian cyst; therefore, vaginal progesterone at a dose of 400 mg twice daily was administered as individualized luteal support and continued until 16 weeks. Although the pregnancy evolved favorably, no causal relationship between progesterone supplementation and the obstetric outcome can be inferred from this single case.

To better understand the clinical presentation, diagnostic approach, and surgical management of adnexal torsion in pregnant versus non-pregnant women, we conducted a comparative review of the literature. Table 2 summarizes the main characteristics of adnexal torsion cases reported in the literature, organized chronologically. It compares pregnant and non-pregnant women in terms of clinical presentation, gestational age, imaging findings, laboratory results, and histological diagnoses. Studies by Bassi et al. and Resapu et al., both published in 2018, have also been included in this comparative analysis [19,20]. The included studies, ranging from 2010 to 2020, show consistent trends in clinical presentation, with abdominal pain being the most frequent symptom across both populations. Pregnant patients often presented earlier and with smaller adnexal masses, particularly in the first trimester, and were more likely to undergo conservative surgical management. Histologically, functional and corpus luteum cysts were more frequent in pregnant patients, whereas dermoid cysts and other benign neoplasms predominated in non-pregnant women. Some studies also reported a higher leukocyte count in pregnant patients, possibly reflecting physiological changes of pregnancy. This comparative review places our observation within the broader published experience but does not constitute a case series. The findings of the present report therefore remain limited by its single-case design and should not be generalized without support from larger studies.

**Table 2:** Comparative characteristics of adnexal torsion in pregnant and non-pregnant women across published studies Comparison of clinical presentation, imaging findings, biological parameters, and histological characteristics of adnexal torsion in pregnant and non-pregnant women across selected studies. WBC: white blood cell count; Doppler: Doppler ultrasound evaluation; OHSS: ovarian hyperstimulation syndrome; ±: standard deviation; not reported: data not available in the original study

Authors / Year	Total Cases	Pregnant Women	Gestational Data (Pregnant)						Non-pregnant Women				
			Cases	Gestational Age (weeks)	Clinical Presentation	Imaging	Biology	Histology	Cases	Clinical Presentation	Imaging	Biology	Histology
Hasson et al. [6]	118	41	41	10.5 ± 6.6	Pain 81.6%, nausea and vomiting 63.2%, tenderness 97.9%	Normal Doppler 61%	White blood cell count 12000 cells/µL	Not reported	77	Pain 85.7%, nausea and vomiting 42%, tenderness 93%	Normal Doppler 45%	White blood cell count 11530 cells/µL	Not reported
Ginath et al. [8]	262	64	64	11.5	Pain 97%, nausea and vomiting 42%, fever 5%	Normal Doppler 70%	White blood cell count 11700 cells/µL	65% benign neoplasms	198	Pain 96%, nausea and vomiting 48%, fever 11%	Normal Doppler 47%	White blood cell count 10400 cells/µL	59% benign tumors
Melcer et al. [9]	227	62	56	Not reported	Pain 100%, nausea and vomiting 75.8%, fever 1.6%	Adnexal mass 41.9%, enlarged ovary 22.6%	White blood cell count >11000 cells/µL	Functional cysts 69.6%	110	Pain 98.2%, nausea and vomiting 54.5%, fever 1.8%	Adnexal mass 61.8%, enlarged ovary 32.7%	White blood cell count >11000 cells/µL	Functional cysts 34.3%
Bassi et al. [19]	1336	163	163	Not reported	Not reported	Not reported	Not reported	Dermoid cyst 18.1%	1173	Not reported	Not reported	Not reported	Dermoid cyst 23.8%
Resapu et al. [20]	76	28	28	1st trimester 73%	Not reported	Not reported	Not reported	Teratoma 14%	48	Nausea and vomiting 42%, mass 13%	Absent vascularity 37%	Not reported	Paraovarian cyst 24%
Seo et al. [15]	239	33	33	Not reported	Pain 90.9%	Not reported	White blood cell count 12090 cells/µL	Corpus luteal cyst 42.4%	206	Pain 85.4%, nausea and vomiting 8.3%	Not reported	White blood cell count 10140 cells/µL	Dermoid cyst 29.6%
Feng et al. [12]	109	25	25	Not reported	Pain 100%, nausea and vomiting 1%	Decreased or absent flow 60%	Not reported	Corpus luteum 40%	84	Pain 90.5%, nausea and vomiting 7%	Decreased or absent flow 69%	Not reported	Mature teratomas 32.1%
Wu et al. [14]	105	33	33	17.3	Pain 93.9%, nausea and vomiting 30.3%	Not reported	White blood cell count 11090 ± 2620 cells/µL	Benign cysts 36.4%	72	Pain 88.9%, nausea and vomiting 44.4%, fever 6.9%	Not reported	White blood cell count 8400 ± 3100 cells/µL	Benign cysts 38.1%

## Conclusions

Adnexal torsion should remain an important differential diagnosis in pregnant patients presenting with acute pelvic pain, even when the associated ovarian cyst is small. Because clinical, laboratory, and imaging findings may be nonspecific, a high index of suspicion and timely surgical evaluation are essential. Conservative management should be prioritized whenever technically feasible to preserve ovarian function, while the surgical approach should be individualized according to the clinical situation, available expertise, and local resources. As this report is based on a single case, its favorable outcome should not be generalized, and the specific contribution of postoperative progesterone supplementation remains uncertain.

## References

[1] Hibbard LT (1985). Adnexal torsion. Am J Obstet Gynecol.

[2] Huchon C, Fauconnier A (2010). Adnexal torsion: a literature review. Eur J Obstet Gynecol Reprod Biol.

[3] Martone S, Troìa L, Luisi S (2021). Adnexal masses during pregnancy: management for a better approach. Gynec Surg.

[4] Practice Committee of the American Society for Reproductive Medicine (2021). Diagnosis and treatment of luteal phase deficiency: a committee opinion. Fertil Steril.

[5] Smorgick N, Pansky M, Feingold M, Herman A, Halperin R, Maymon R (2009). The clinical characteristics and sonographic findings of maternal ovarian torsion in pregnancy. Fertil Steril.

[6] Hasson J, Tsafrir Z, Azem F (2010). Comparison of adnexal torsion between pregnant and nonpregnant women. Am J Obstet Gynecol.

[7] Houry D, Abbott JT (2001). Ovarian torsion: a fifteen-year review. Ann Emerg Med.

[8] Ginath S, Shalev A, Keidar R, Kerner R, Condrea A, Golan A, Sagiv R (2012). Differences between adnexal torsion in pregnant and nonpregnant women. J Minim Invasive Gynecol.

[9] Melcer Y, Sarig-Meth T, Maymon R, Pansky M, Vaknin Z, Smorgick N (2016). Similar but different: a comparison of adnexal torsion in pediatric, adolescent, and pregnant and reproductive-age women. J Womens Health (Larchmt).

[10] Niafar F, Mirfendereski S, Rostamzadeh A (2014). Diagnostic efficacy of sonography for diagnosis of ovarian torsion. Zahedan Journal of Research in Medical Sciences.

[11] Wattar B, Rimmer M, Rogozinska E, Macmillian M, Khan KS, Al Wattar BH (2021). Accuracy of imaging modalities for adnexal torsion: a systematic review and meta-analysis. BJOG.

[12] Feng JL, Zheng J, Lei T, Xu YJ, Pang H, Xie HN (2020). Comparison of ovarian torsion between pregnant and non-pregnant women at reproductive ages: sonographic and pathological findings. Quant Imaging Med Surg.

[13] Jalal M, El Qasseh R, Youssouf N (2022). Healthy adnexal torsion in pregnancy: a case report. Int J Surg Case Rep.

[14] Wu WF, Wang ZH, Xiu YL, Xie X, Pan M (2020). Characteristics and surgical invervention of ovarian torsion in pregnant compared with nonpregnant women. Medicine (Baltimore).

[15] Seo SK, Lee JB, Lee I, Yun J, Yun BH, Jung YS, Chon SJ (2019). Clinical and pathological comparisons of adnexal torsion between pregnant and non-pregnant women. J Obstet Gynaecol Res.

[16] Dvash S, Pekar M, Melcer Y, Weiner Y, Vaknin Z, Smorgick N (2020). Adnexal torsion in pregnancy managed by laparoscopy is associated with favorable obstetric outcomes. J Minim Invasive Gynecol.

[17] Hatata MA, Soliman DA, Hosni KA (2023). Approach to ovarian torsion with corpus luteum re-moval in early pregnancy. Qatar Med J.

[18] Coomarasamy A, Devall AJ, Brosens JJ (2020). Micronized vaginal progesterone to prevent miscarriage: a critical evaluation of randomized evidence. Am J Obstet Gynecol.

[19] Bassi A, Czuzoj-Shulman N, Abenhaim HA (2018). Effect of pregnancy on the management and outcomes of ovarian torsion: a population-based matched cohort study. J Minim Invasive Gynecol.

[20] Resapu P, Rao Gundabattula S, Bharathi Bayyarapu V, Pochiraju M, Surampudi K, Dasari S (2019). Adnexal torsion in symptomatic women: a single-centre retrospective study of diagnosis and management. J Obstet Gynaecol.

